# Identification of seven hypoxia-related genes signature and risk score models for predicting prognosis for ovarian cancer

**DOI:** 10.1007/s10142-022-00956-3

**Published:** 2023-01-16

**Authors:** Yan Huang, Yuqi Zhou, Meiqin Zhang

**Affiliations:** 1 Department of Gynecologic Oncology, Fudan University Shanghai Cancer Center, Shanghai, 200000 China; 2 Department of Oncology, Shanghai Medical College Fudan University, Shanghai, 200000 China

**Keywords:** Ovarian cancer, Hypoxia, TIME, Prognosis, Biomarkers

## Abstract

Ovarian cancer (OC) is the most common malignant cancer in the female reproductive system. Hypoxia is an important part of tumor immune microenvironment (TIME), which is closely related to cancer progression and could significantly affect cancer metastasis and prognosis. However, the relationship between hypoxia and OC remained unclear. OCs were molecularly subtyped by consensus clustering analysis based on the expression characteristics of hypoxia-related genes. Kaplan–Meier (KM) survival was used to determine survival characteristics across subtypes. Immune infiltration analysis was performed by using Estimation of Stromal and Immune cells in Malignant Tumors using Expression data (ESTIMATE) and microenvironment cell populations-counter (MCP-Counter). Differential expression analysis was performed by using limma package. Next, univariate Cox and least absolute shrinkage and selection operator (LASSO) regression analyses were used to build a hypoxia-related risk score model (HYRS). Mutational analysis was applied to determine genomic variation across the HYRS groups. The Tumor Immune Dysfunction and Exclusion (TIDE) algorithm was used to compare the effectiveness of HYRS in immunotherapy prediction. We divided OC samples into two molecular subtypes (C1 and C2 subtypes) based on the expression signature of hypoxia genes. Compared with C1 subtype, there was a larger proportion of poor prognosis genotypes in the C2 subtype. And most immune cells scored higher in the C2 subtype. Next, we obtained a HYRS based on 7 genes. High HYRS group had a higher gene mutation rate, such as TP53. Moreover, HYRS performed better than TIDE in predicting immunotherapy effect. Combined with clinicopathological features, the nomogram showed that HYRS had the greatest impact on survival prediction and a strong robustness.

**Supplementary Information:**

The online version contains supplementary material available at 10.1007/s10142-022-00956-3.

## Introduction

Ovarian cancer (OC) is the third most common malignant tumor of the female reproductive system, with an incidences lower than cervical cancer and uterine corpus cancer (Jayson et al. 2014). OC occurrence ranks the seventh among all female cancer patients (Reid and Permuth 2017). Although its incidence is not the highest, the mortality and prognosis of OC are significantly poor (Caan and Thomson 2007). The death rate of OC will be even higher in 2040, according to statistical models (Kamath Mulki 2021). Asymptomatic, secret growth, and lack of screening are important reasons for the late diagnosis of OC (Jacobs et al. 2004). Therefore, OC is also known as the “silent killer” (Das and Bast 2008). The current main treatments for OC are surgery and cisplatin chemotherapy. Although the rise of immunotherapy, targeted therapy, and other therapeutic methods has advanced the treatment of OC, the improvement of 5-year survival rate is still slow (Lee et al. 2018). The limitations of OC therapy have hindered the clinical OC-targeted therapy; therefore, novel prognostic models and loci to make targeted therapy more feasible are urgently needed.

Hypoxia, which is an important feature of solid tumors like OC, could result in increased patient resistance to therapy due to reduced oxygen availability, ultimately favoring tumor progression (Brahimi-Horn and Chiche 2007). Disruption of cell cycle checkpoints and reversal of oncogene/suppressor genes are considered to be the initial stages of tumorigenesis (Molinari 2000; Moron et al. 2018; Duffy and Crown 2021). Moreover, hypoxia is one of the foundations to tumor progression, stronger drug resistance, and metastasis (Vaupel et al. 2007; Wang et al. 2021b). Hypoxia has been reported to contribute to the development of many different cancers such as OC and resistance to platinum-based chemotherapy drugs (Selvendiran et al. 2009). Previous studies suggested that hypoxia conferred cisplatin resistance by interfering the expression of L1 cell adhesion molecule (L1-CAM), signal transducer and activator of transcription 3 (STAT3), and P53 (Selvendiran et al. 2009; Stoeck et al. 2007; Graeber et al. 1996). In OC, the relationship between cisplatin resistance and hypoxia has been reported, and angiopoietin-like 4 (ANGPTL4) was identified to be a potential biomarker for OC targeting therapy site (McEvoy et al. 2015). However, the discovery of more treatment sites would be beneficial to improve OC treatment.

Here, we assessed 200 hypoxia-related genes expressed in OC and divided OC into two distinct molecular subtypes based on hypoxia-related genes associated with OC prognosis. Kaplan–Meier survival analysis showed that the C1 subtype had a favorable prognosis. We also compared in detail the immune profile and susceptibility to treatment among different subtypes. Moreover, seven hypoxia-related gene signatures were obtained to build an effective risk score model, including SNRPD1, KLF4, UQCRFS1, KRAS, HOXA5, ISG20, and GMPR.

## Methods and materials

### Data sources

The gene expression profiles of OC were downloaded from The Cancer Genome Atlas Program (TCGA) public database (https://www.cancer.gov/about-nci/organization/ccg/research/structural-genomics/tcga). For TCGA cohort data, first samples without clinical information were removed, followed by those without survival state and survival time. Further, the filtering time was set to be shorter than 30 days and more than 10 years. Three hundred fifty-four cases of tumor samples were finally included in research. Moreover, we downloaded 153 samples in Gene Expression Omnibus (GEO) public database (Vathipadiekal et al. 2015). For GSE cohort data, first samples without clinical information were removed, and then samples where the survival state and lack of survival time also were removed. Next, ENSEMBL ID was converted to Gene Symbol.

### Appraisal hypoxia-related molecular subtype

The hypoxia-related genes were derived from the Molecular Signatures Database (MSigDB) database of the hypoxic channel “HAllMark_hypoxia,” with a total of 200 genes (Liberzon et al. 2011).

ConsensusClusterPlus (R Bioconductor/R package, v1.60.0) (Wilkerson and Hayes 2010) was used to perform consistency cluster analysis and identify unique molecular subtypes. Eighty percent samples were carried out 500 bootstraps using km algorithm and distance of 1 Pearson correlation. The number of clusters (*K*) was 2 to 10. The optimal classification was determined by calculating the consistency matrix and cumulative distribution function (CDF) of the consistency, and molecular subtype of the samples was obtained.

### Immune infiltration analysis

The Estimation of Stromal and Immune cells in Malignant Tumors using Expression data (ESTIMATE) algorithm was used to assess immune infiltration (Yoshihara et al. 2013), and microenvironment cell populations-counter (MCP-Counter) software and single sample gene set enrichment analysis (ssGSEA) function of GSEA software were selected to analyze the scores of 10 immune cells and 28 immune cells, respectively (Charoentong et al. 2017).

### Differential expression analysis

Limma (R Bioconductor/R package, v3.52.3) is selected for differentially expressed genes (DEGs) screening and we set |log2 FoldChange|> 2 and *p*-value < 0.05 as the threshold (Smyth 2005).

### Function enrichment analysis

DEGs were performed with Kyoto Encyclopedia of Genes and Genomes (KEGG) pathway enrichment analysis by using GSEA software (Reimand et al. 2019). The background gene set was c2.cp.kegg.v7.0.symbols.gmt. *p*-value < 0.05 was considered as significant enrichment.

### Hypoxia-related risk model (HYRS) construction

The condition of screening prognostically significantly gene associated with hypoxia-related phenotypes was determined using univariate Cox analysis under *p*-value < 0.05. Next, least absolute shrinkage and selection operator (LASSO) regression analyses were used to reduce the candidate genes. The risk score for each patient was calculated using the following formula:

$$\mathrm{HYRS}=\Sigma\beta i\times\mathrm{Expi}$$

*i* refers to the expression level of key genes in the prognosis of hypoxia-related phenotype, and *β* is the Cox regression coefficient of the corresponding gene. According to the threshold of “0,” the patients were divided into high and low risk groups of HYRS. The survival curve was drawn by the Kaplan–Meier method for prognostic analysis, and the log-rank test was used to determine the significant difference.

### Tumor mutation analysis

Genomic variation information is from previous study (Thorsson et al. 2018). MutationalPatterns (R package) was used to analyze mutation signatures (Blokzijl et al. 2018). Aneuploidy score and homologous recombination between different HYRS groups were compared. In addition, mutational signatures of tumor genes were displayed using waterfall plots.

### Predicting response to immunotherapy

The TIDE algorithm was used to validate the effect of HYRS in predicting clinical responsiveness to immune checkpoint inhibitors (ICI) (Jiang et al. 2018). The TIDE algorithm evaluates three cell types that limit T cell infiltration into tumors, including tumor-associated macrophages (TAMs), myeloid-derived suppressor cells (MDSCs), the M2 subtype of tumor-associated fibroblasts (CAFs), and two different mechanistic tumor immune escape scores, including immunosuppressive factor rejection of CTLs score and tumor-infiltrating cytotoxic T lymphocytes (CTLs) dysfunction score (dysfunction).

### HYRS improvement and survival prediction

To quantify risk assessment and survival possibility in OC patients, we combined HYRS and other clinicopathological features to develop a nomogram by using forsetplot (R package, v3.1.0) (https://gforge.se/packages/). Next, calibration curve was selected to evaluate the accuracy of the model. In addition, decision curve was used to evaluate the reliability of the model.

### Statistical analysis

R (4.0.2) software was used for statistical analysis. Wilcoxon nonparametric rank sum test was used to analyze the differences. *p* < 0.05 was considered to be statistically significant. Sangerbox was used for analysis (Shen et al. 2022).

## Results

### Identification of molecular subtypes associated with hypoxia prognosis

**Fig. 1:**
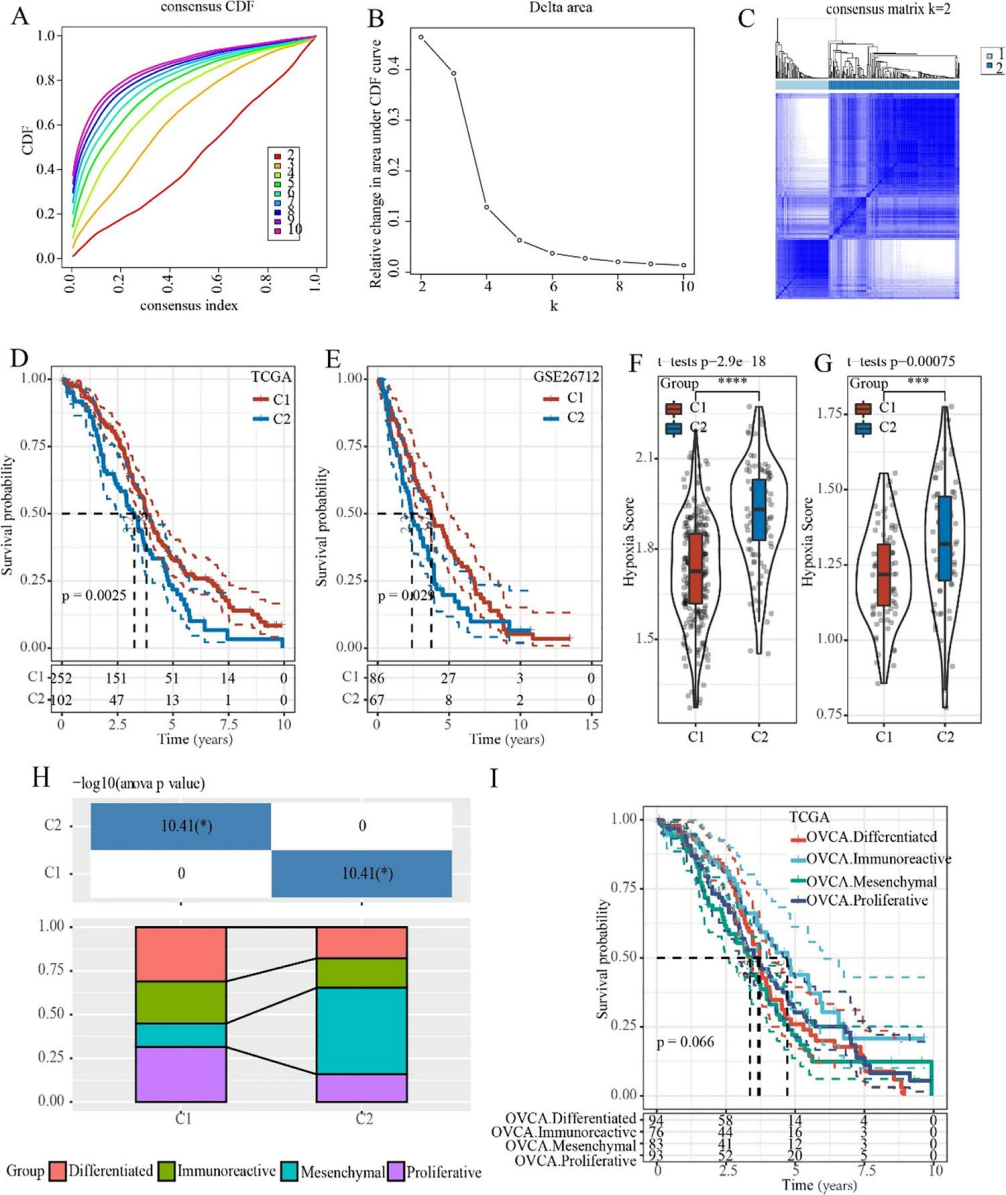
Identification of hypoxia-related ovarian cancer subtypes. **A** The cumulative distribution function (CDF) curve in TCGA cohort. **B** The CDF Delta area curve in TCGA cohort. **C** Heat map of sample clusters when consensus *k* = 2 in the TCGA cohort. **D** C1 had longer overall survival (OS) than that in C2 in the TCGA cohort. **E** C1 had a better survival outcome in the GSE cohort. **F** Differences in hypoxia scores among subtypes in the TCGA cohort. **G** Differences in hypoxia scores among subtypes in the GSE cohort. **H** Molecular subtype comparison information. **I** Survival curves of reported molecular subtypes. *p < 0.05, **p < 0.01, ***p < 0.001, ****p < 0.0001

A total of 200 genes related to hypoxia were subjected to univariate regression Cox analysis in the TCGA dataset, and 14 genes related to prognosis were finally obtained (*p*-value < 0.05, Table S1). Next, 14 hypoxia-signature genes were used to perform consensus clustering analysis. According to the CDF to determine the optimal number of clusters, and the CDF Delta area curve showed that when Cluster was selected as 2, the clustering result was relatively stable (Fig. 1A and B). Two hypoxia-related molecular subtypes were shown in a heat map (Fig. 1C). Kaplan–Meier survival analysis in the TCGA and GSE cohorts demonstrated that the C1 subtype had a better prognosis, while the C2 subtype had a poorer prognosis (Fig. 1D and E). Hypoxia scores suggested that the C2 subtype had higher hypoxia scores both in the TCGA and GSE cohorts (Fig. 1F and G). Moreover, previous study classified OC into four categories based on gene expression profiles, which were compared with the two molecular subtypes of this study. And the results suggested that the previously reported mesenchymal subtype with the worst prognosis had the largest proportion of the C2 subtype (Fig. 1H and I).

### Immune signatures in C1 and C2 subtypes

**Fig. 2:**
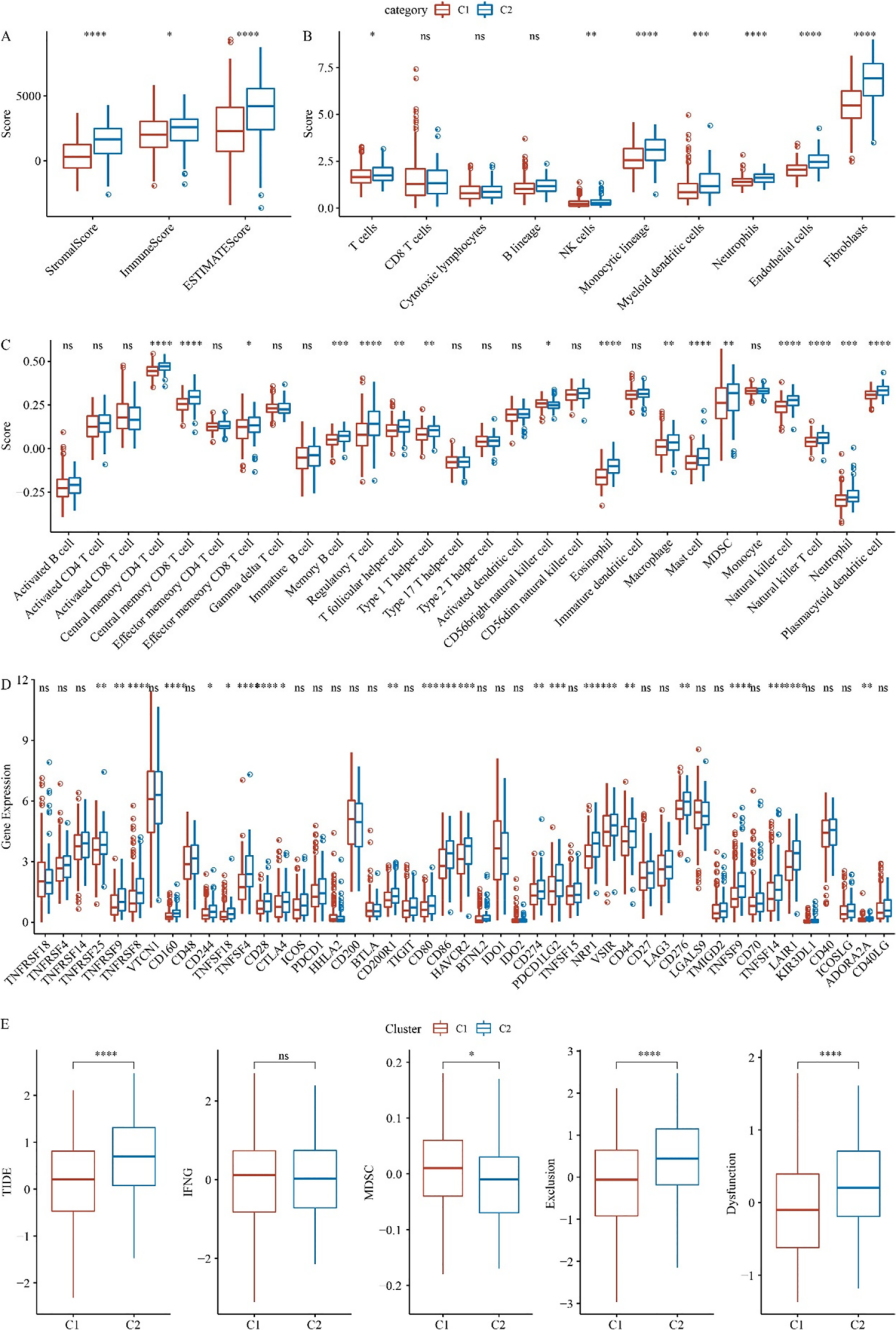
Immune signatures in different molecular subtypes. **A** Differences in immune infiltration among molecular subtypes in the TCGA cohort. **B** Score differences of 10 immune cells among molecular subtypes were analyzed by MCP-Counter. **C** ssGSEA analysis of 28 immune cells scores among molecular subtypes. **D** Differences in immune checkpoint gene expression between C1 and C2 subtypes in the TCGA cohort. **E** Differences in TIDE scores between C1 and C2 subtypes in the TCGA cohort. *p < 0.05, **p < 0.01, ***p < 0.001, ****p < 0.0001

Differences in the tumor immune microenvironment (TIME) are critical to tumor progression, especially in tumor prognosis and metastasis. The ESTIMATE algorithm was used to assess immune cell infiltration, and the results demonstrated that the C2 subtype had a significantly higher immune score, Stromal score, and ESTIMATE score (Fig. 2A). The immune scores of different types of immune cells showed that the immune scores of most cells were higher in the C2 subtype, such as endothelial cells and fibroblasts, T cells, monocytic lineage, NK cells, neutrophils, and myeloid dendritic cells (Fig. 2B). The ssGSEA algorithm was used to assess the infiltration of 28 immune cells, and the degree of immune infiltration of most cells was higher in the C2 subtype, such as central memory CD4 T cells (Fig. 2C). Abnormal expression of cellular immune checkpoints can promote tumor progression. Differential expression analysis of 47 immune checkpoint-related genes demonstrated that the expression of 23 immune checkpoint-related genes was significantly higher in C2 than in C1 subtype (Fig. 2D). The results of the TIDE algorithm examining the response of two subtypes to immunotherapy showed that the TIDE score, exclusion score, and dysfunction score of the C2 subtype were higher than the C1 subtype, while MDSC score was lower in C2 (Fig. 2E).

### Identification of DEGs in different hypoxia-associated molecular subtypes

**Fig. 3:**
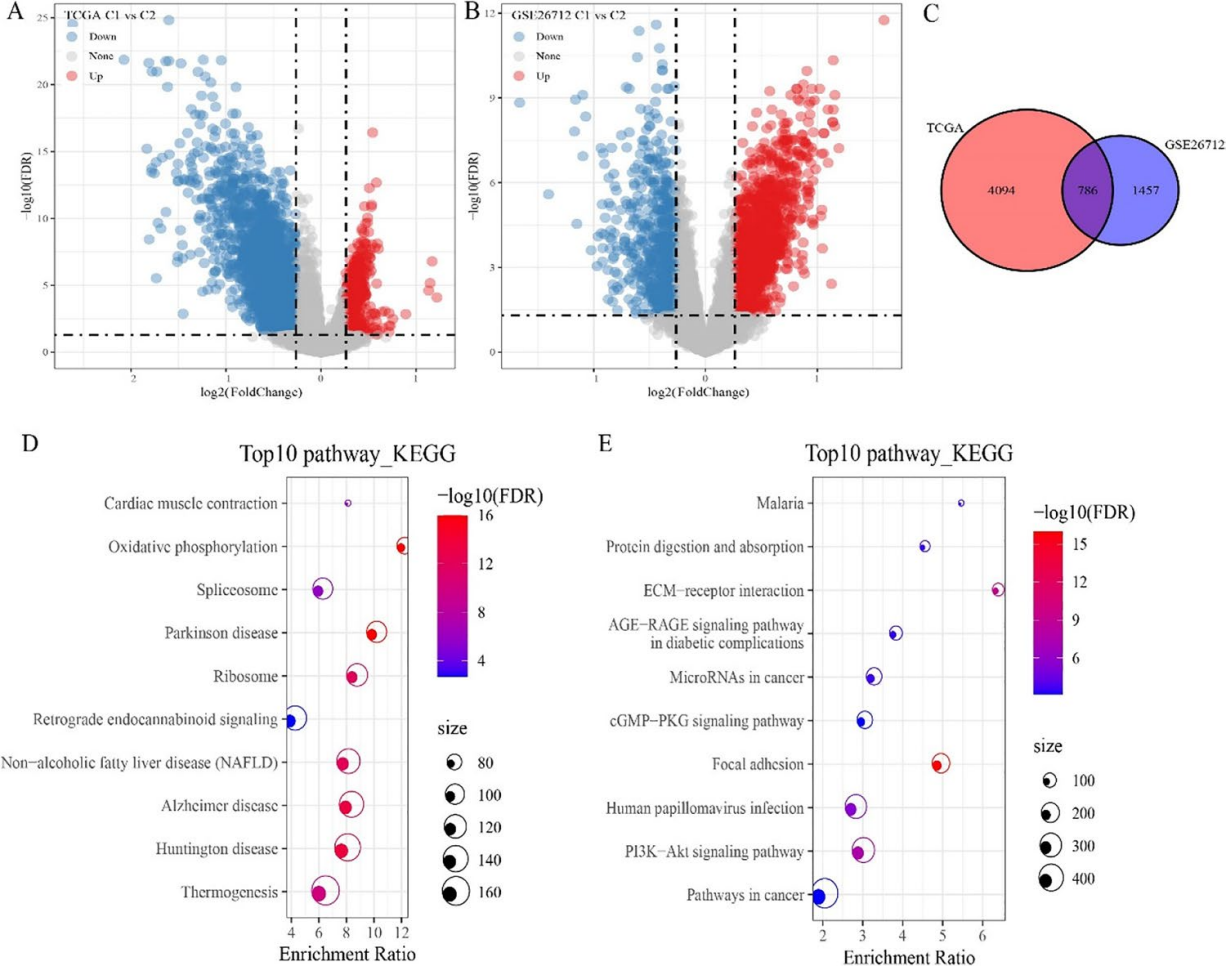
Identification of differentially expressed genes between C1 and C2 subtypes. **A** Volcano plot showed differential expressed genes (DEGs) between C1 and C2 subtypes in the TCGA cohort. **B** Volcano plot showed DEGs between C1 and C2 subtypes in the GSE cohort. **C** Venn diagram of DEGs of the intersection of TCGA and GSE26712. **D** KEGG pathways of up-regulated DEGs. **E** KEGG pathways of down-regulated DEGs

Different gene transcriptional states tend to be associated with different cellular states; therefore, we performed gene differential expression analysis. In the TCGA cohort, we obtained 4880 DEGs, of which 520 genes were up-regulated in C1 and 4360 genes were down-regulated in C1 (Fig. 3A). In the GSE cohort, 2243 DEGs were obtained, of which 1585 genes were up-regulated expression in C1 subtype, and 658 genes were down-regulated in C1 (Fig. 3B). Further intersecting the DEGs between the two cohorts filtered 211 shared up-regulated DEGs and 524 shared down-regulated DEGs. We also compared the total DEGs and found 786 DEGs in common between the two cohorts (Figs. 3C). The top 10 KEGG pathway demonstrated that the shared up-regulated DEGs were involved in regulating oxidative phosphorylation (Fig. 3D). The down-regulated DEGs were involved in regulating PI3K-Akt signaling pathway (Fig. 3E).

### Establishment of a hypoxia-related risk score model (HYRS)

**Fig. 4:**
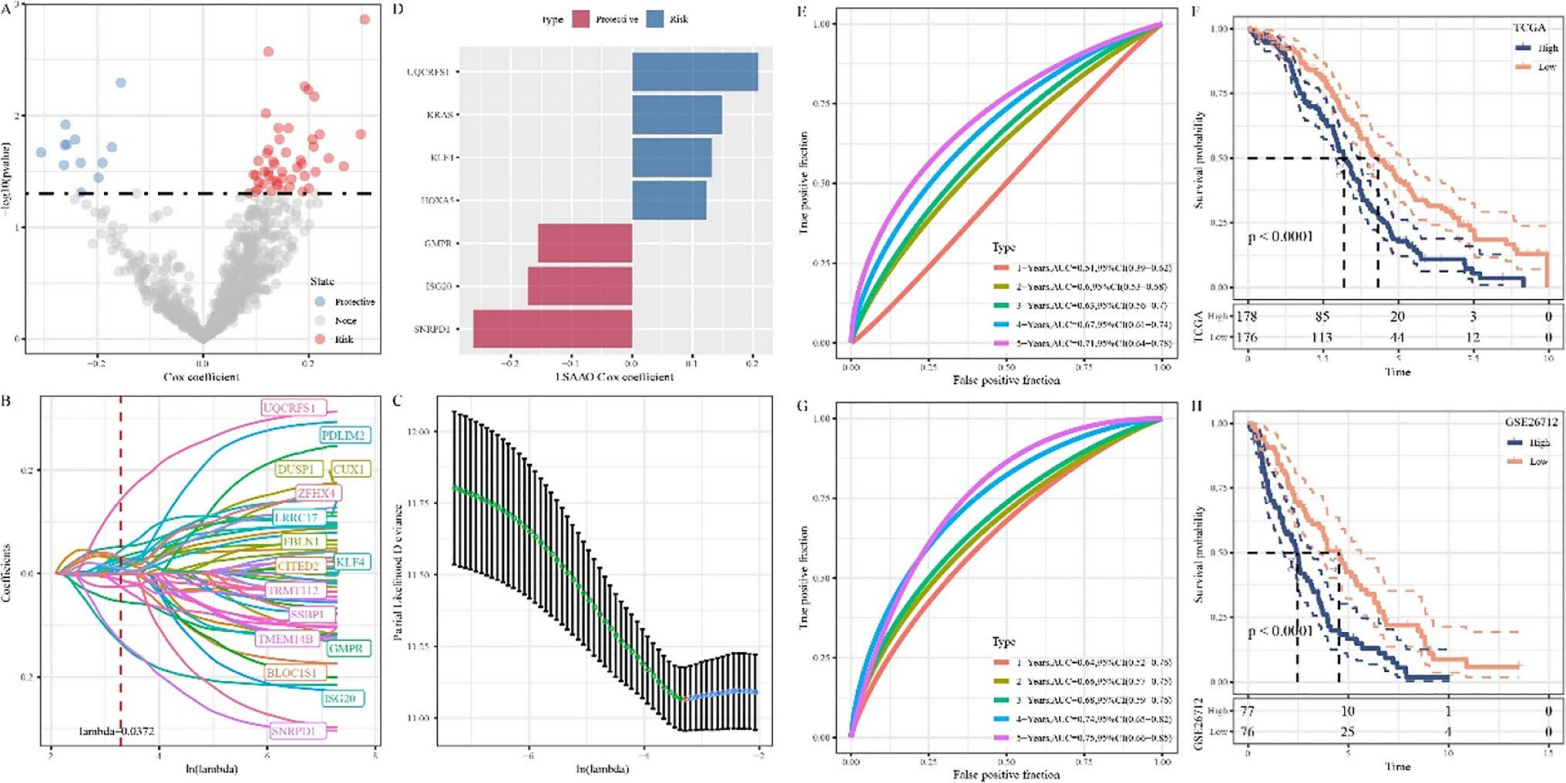
Establishment of a hypoxia-related risk scoring model. **A** Volcano plot showing candidate signature genes associated with OC prognosis. **B** Trajectories of candidate genes as lambda changes. **C** Confidence interval under lambda. **D** Distribution of LASSO coefficients of the hypoxia-related gene signature. **E** Receiver operating characteristic (ROC) curve of HYRS in TCGA cohort. **F** Survival curve of the high HYRS and low HYRS groups in TCGA cohort. **G** ROC curve of HYRS in GSE cohort. **H** Survival curve of the high HYRS and low HYRS groups in GSE cohort

Univariate Cox regression analysis of 786 shared DEGs determined 59 survival-related genes, including 47 “Risk” genes and 12 “Protective” genes (Fig. 4A). LASSO regression analysis was used to further identify key prognostic genes, and then tenfold cross-validation was used for model construction. It was found that the model was optimal when lambda = 0.0372 (Fig. 4B and C). Finally, 7 genes were obtained as key genes affecting prognosis, including UQCRFS1, KRAS, KLF4, HOXA5, GMPR, ISG20, and SNRPD1 (Fig. 4D). Hypoxia-related risk models were further developed as follows: HYRS = (− 0.35*SNRPD1) + 0.117*KLF4 + 0.235*UQCRFS1 + 0.158*KRAS + 0.09*HOXA5 + (− 0.217*ISG20) + (− 0.103*GMPR). In TCGA cohort, ROC curve analysis demonstrated that HYRS had effective prediction efficiency (5 years AUC = 0.71) (Fig. 4E). Kaplan–Meier survival analysis showed that the low HYRS score group had a better prognosis (Fig. 4F). In GSE cohort, ROC curve showed the 5-year AUC = 0.75 (Fig. 4G). As expected, the Kaplan–Meier survival analysis indicated that the low HYRS score group had also a better prognosis (Fig. 4H).

### Clinical characteristics among different HYRS groups

**Fig. 5:**
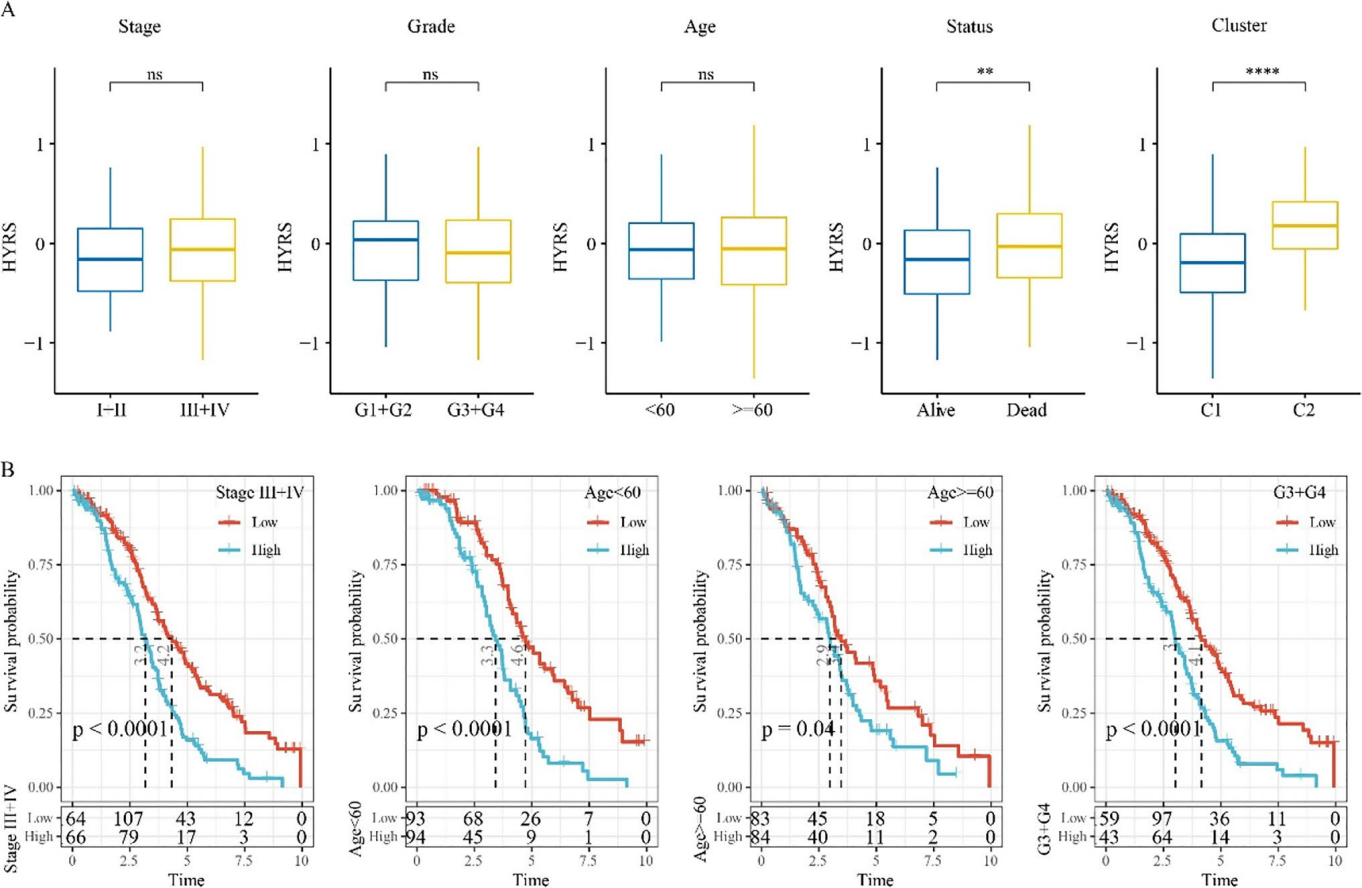
Clinical characteristics in different HRYS groups. **A** Differences in HYRS between different clinicopathological groups in the TCGA cohort, following by stage, grade, age, status, and cluster. **B** Survival curves between the high and low HYRS groups, which were divided based on clinicopathological patients in the TCGA cohort

Analysis of HYRS scores for different clinical features showed no differences, including stage, grade, and age (Fig. 5A). However, the dead samples had higher HYPS in comparison to alive samples. High HYRS scores were mostly dead. Moreover, C2 subtypes had a higher HYRS score (Fig. 5A). OC samples with clinical characteristics, including stages III–IV, age < 60, age ≥ 60, and grade 3 + grade 4, were divided into two HYRS groups, and Kaplan–Meier survival analysis showed that the low HYRS groups had a better survival outcome in comparison to the high HYRS group. Specifically, among the different clinical feature groups, the low HYRS group had a better prognosis (Fig. 5B).

### Mutation characteristics in the high HYRS and low HYRS groups

**Fig. 6:**
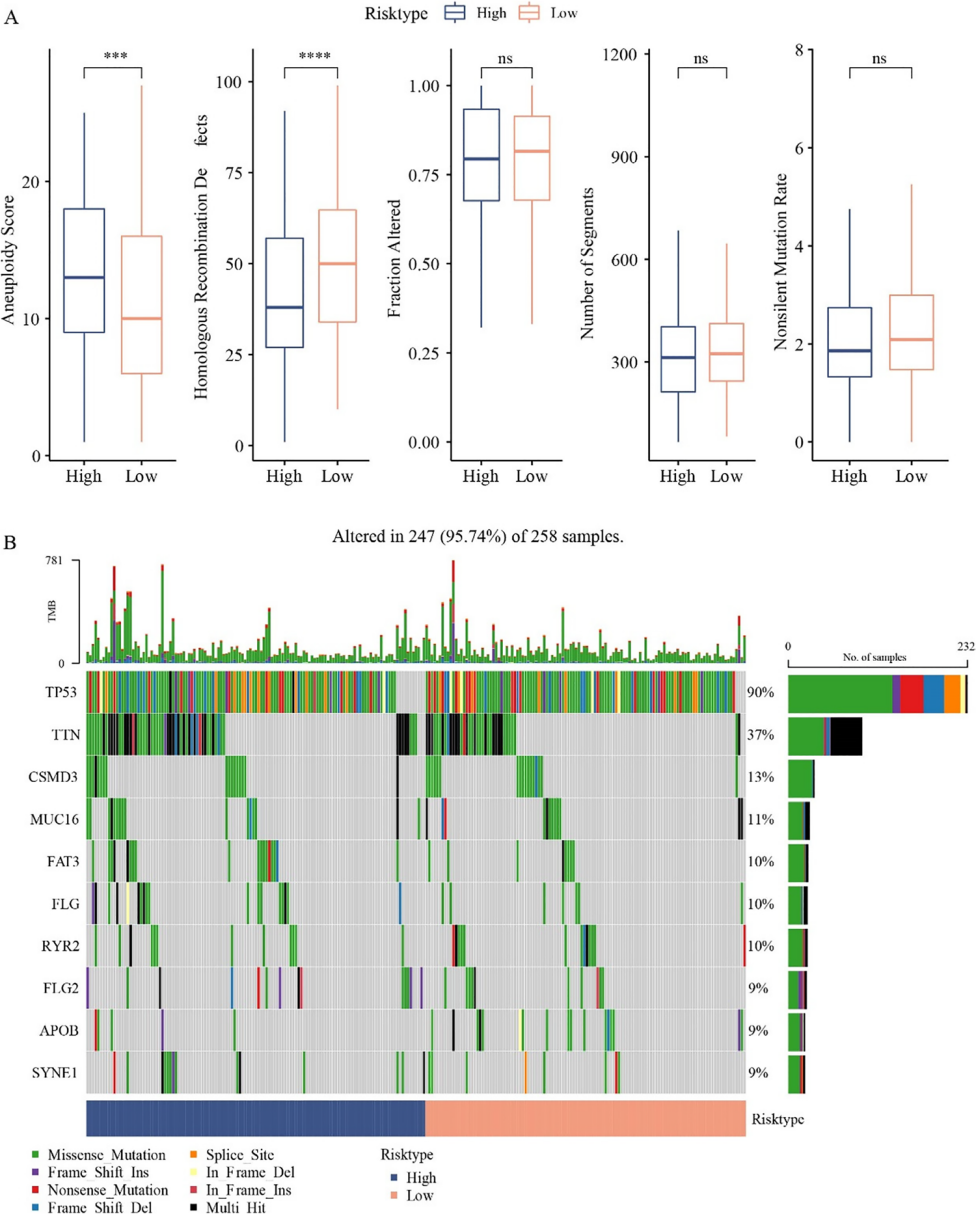
Genomic mutations in different HYRS subgroups in the TCGA cohort. **A** Comparison of homologous recombination defects, aneuploidy score, fraction altered, number of segments, and nonsilent mutation rate in the high group and low group (Wilcoxon test, *p < 0.05; **p < 0.01; ***p < 0.001; and ****p < 0.0001). **B** Somatic mutation in the high group and low group

As gene mutations could increase the risk of carcinogenesis, we analyze differences in genomic alterations between the high HYRS and low HYRS groups. The mutation characteristics of TCGA cohorts showed that the high HYRS groups had high aneuploidy score and homologous recombination defects; however, the fraction altered, number of segments, and nonsilent mutation rate showed no significance (Fig. 6A). Gene mutation analysis of the high HYRS and low HYRS groups demonstrated the gene mutation of TP53, TTN, and CSMD3 was higher in the high HYRS groups (Fig. 6B).

### Pathway differences in the high HYRS and low HYRS groups

**Fig. 7:**
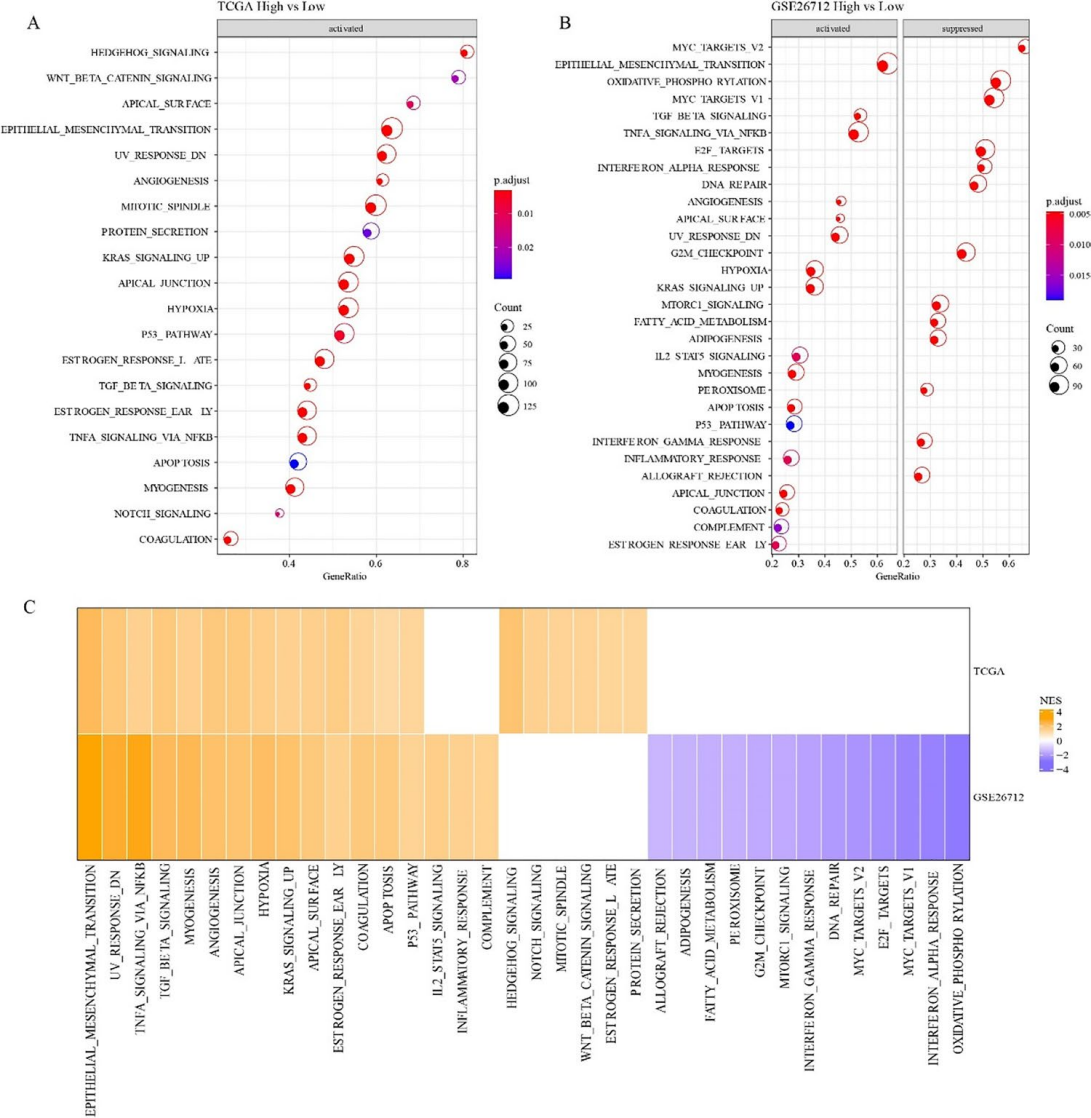
Pathway differences in different HRYS groups. **A** KEGG pathway in the high *vs* low HYRS group in TCGA cohort. **B** KEGG pathway in the high *vs* low HYRS group in GSE cohort. **C** Comparative analysis of metabolic pathway differences in TCGA and GSE cohorts

Differences in expression of gene pathways in cancer may be the key leading to different prognoses. GSEA results suggested that in the TCGA cohort, compared with the low HYRS group, 20 pathways were activated in the high group, such as HEDGEHOG_SIGNALING and WNT_BETA_CATENIN_SIGNALING (Fig. 7A); in the GSE26712 cohort, 17 pathways were activated, like EPITHELIAL_MESENCHYMAL_TANSTION and TNFA_SIGNALING_VIA_NFKB (Fig. 7B). Moreover, TNFA_SIGNALING_VIA_NFKB, P53_PATHWAY, and TGF_BETA_SIGNALING were activated both in TCGA and GSE cohorts (Fig. 7C).

### Efficacy assessment of HYRS in immunotherapy

**Fig. 8:**
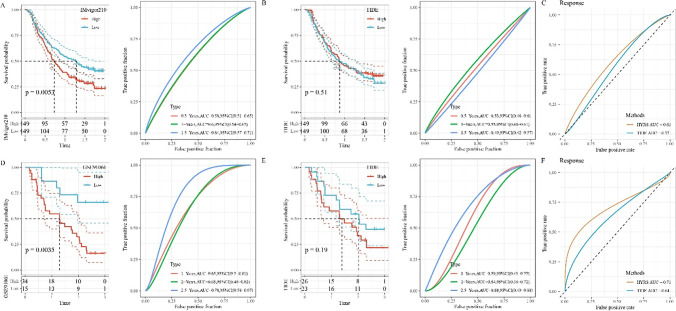
Efficiency of HYRS model. **A** Survival and ROC curves of the high HYRS and low HYRS groups in the IMvigor210 cohort. **B** Survival and ROC curves of the high TIDE and low TIDE groups in the IMvigor210 cohort. **C** ROC curve of the HRYS group and TIDE group in IMvigor210 cohort. **D** Survival and ROC curves of the high HYRS and low HYRS groups in the GSE91061 cohort. **E** Survival and ROC curves of the high TIDE and low TIDE groups in the GSE91061 cohort. **F** ROC curve of the HRYS group and TIDE group. **D** ROC curve of the HRYS group and TIDE group in GSE91061 cohort

The development of immunotherapy has brought hope to OC patients; therefore, we evaluated the effectiveness of HRYS in the prognosis of immunotherapy by comparing with the TIDE algorithm. In IMvigor210 cohorts, the low HRYS groups had better prognosis and a 5-year AUC = 0.64 were observed (Fig. 8A); however, there was no significant difference in prognosis among different groups of TIDE and 5-year AUC = 0.49 (Fig. 8B). The AUC of HYRS and TIDE for immunotherapy effect showed the AUC of HYRS was higher (Fig. 8C). To avoid test results, we selected the GSE91061 cohort for further validation, and found that the low HRYS groups had better prognosis and 5-year AUC = 0.78 (Fig. 8D). Similarly, the TIDE groups were still not significantly different (*p*-value = 0.19) (Fig. 8E). The AUC of HYRS and TIDE for immunotherapy effect showed similar results compared to IMvigor210 cohorts (Fig. 8F).

### HYRS combines clinicopathological features to further improve prognostic models and survival prediction

**Fig. 9:**
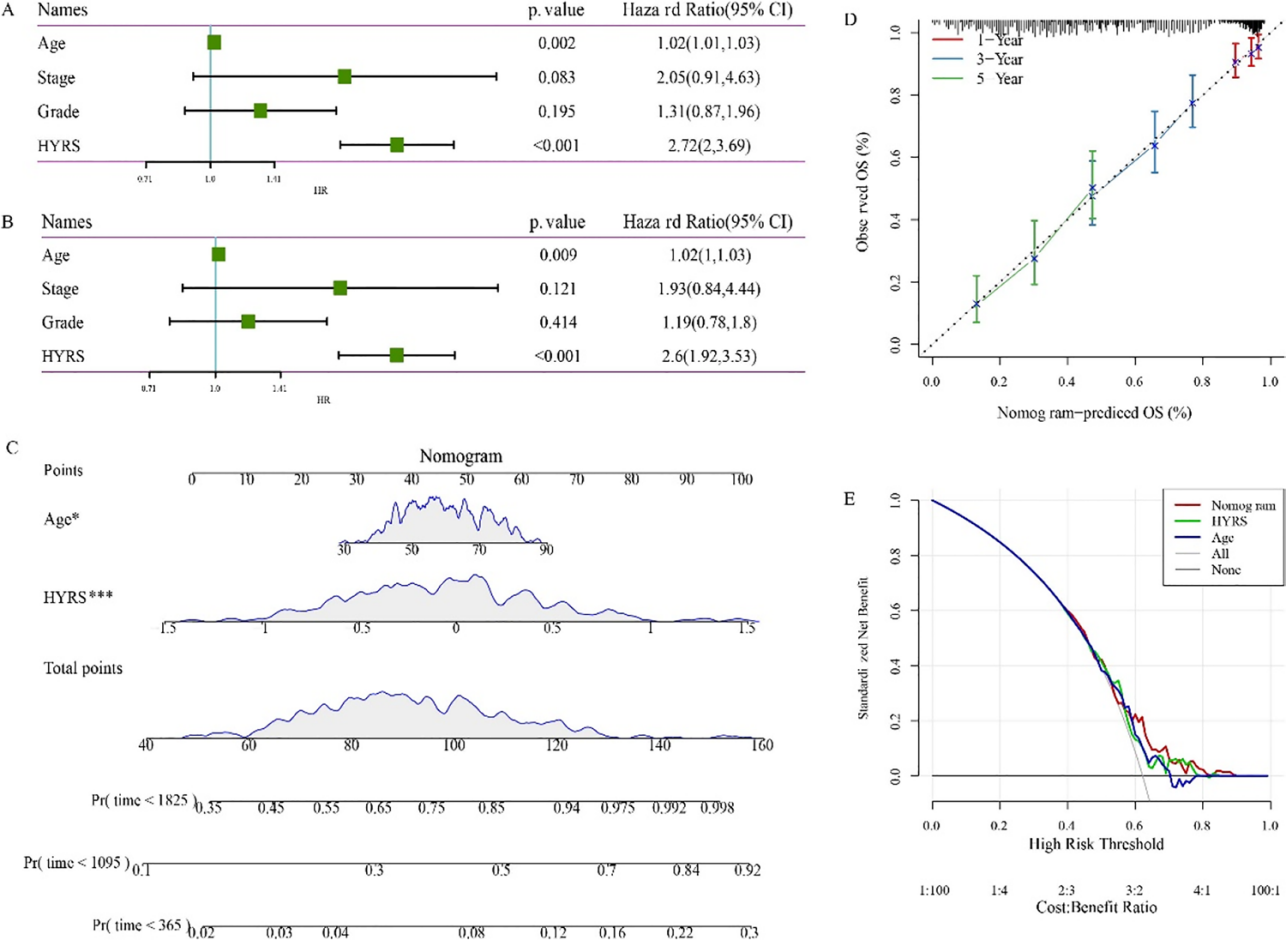
Improvements in prognostic models and survival prediction. **A** Univariate Cox regression analysis of HYRS and clinicopathological features. **B** Multivariate Cox regression analysis of HYRS and clinicopathological features. **C** Nomogram model combined by HYRS and age. **D** 1-, 3-, and 5-year calibration curves of the nomogram. **E** The decision curve of the nomogram

Univariate and multivariate Cox regression analysis of HYRS and clinicopathological features revealed that age and HYRS were independent prognostic factors (Fig. 9A and B). To quantify the risk assessment and survival probability of OC patients, we combined HYRS and age to build a nomogram. From the model results, the assembled nomogram had the greatest impact on predicting prognosis (Fig. 9C). The calibration curve was used to evaluate the prediction accuracy of the model. The results showed that the predicted calibration curve for the three calibration points at 1, 3, and 5 years nearly overlapped with the standard curve (Fig. 9D). The decision curve was used to assess the reliability of the model, and the results showed that both HYRS and nomogram had significantly higher benefits than the extreme curves, and that both the two showed the strongest survival predictors compared with other clinicopathological features (Fig. 9E).

## Discussion

OC is the third most malignant cancer of the female reproductive system and the eighth most lethal of all female cancers (Bray et al. 2018). OC treatment options have been improved significantly over the decades with advances in surgical techniques, at the same time, the advent of new and effective drugs can extend patients’ life expectancy; however, metastasis and recurrence are the leading causes of death (O’Malley. 2019). The use of age and pathological staging to predict the prognosis of OC has been used in clinical practice, but the accuracy is low due to the large variability of individual patient factors. The rise of immunotherapy has brought new insights into OC therapy (Kandalaft and Odunsi 2020). The hypoxia signature in TIME plays a crucial role in immunotherapy and cancer development (Brahimi-Horn and Chiche 2007). Therefore, biomarkers related to hypoxia should be studied urgently. Here, in this study, the OC data in the TCGA and GSE databases were accurately classified according to hypoxia-related genes, and further survival analysis, clinical feature analysis, construction of HYRS model, mutation analysis, immune feature analysis, etc. were used to confirm the OC-related hypoxia characteristics.

Hypoxia is an important feature in the local microenvironment of tumor tissue. Reduced availability of oxygen greatly increases patient resistance to therapy and favors tumor progression. Moreover, hypoxia induces the expression of many genes responsible for increased tumor invasion and metastasis, resulting in deranged gene expression (Chan et al. 2007; Wang et al. 2021a). Univariate Cox regression analysis obtained 14 hypoxia-signature genes associated with the prognosis of OC, and then based on the expression profiles of hypoxia-signature genes, we segmented OC into two distinct molecular subtypes (C1 and C2 subtypes) using ConsensusClusterPlus. A previous study reported four molecular subtypes in OC according to all gene expression levels (Thorsson et al. 2018). Comparative analysis showed that the mesenchymal subtype with the worst prognosis in the previous study accounted for the largest proportion of the C2 subtypes obtained by our clustering, and the Kaplan–Meier survival analysis showed that the C2 subtype had a worse prognosis than the C1 subtype, suggesting that our hypoxia-related molecular subtype had a better indication. The main difference was that we identified molecular subtypes based on hypoxia-related genes and confirmed significant differences among subtypes from multiple dimensions, which can be considered as a classification for clinical patients and then for precision treatment.

Immune cell infiltration is closely related to cancer development and prognosis, specifically, accumulation of tumor-infiltrating lymphocytes predicts increased survival, while increases in immunosuppressive regulatory T cells are associated with poor prognosis (Santoiemma 2015). As expected, T cells, NK cells, etc. were significantly increased in C2 subtypes, as shown by ssGSEA and MCP-Counter. The TIDE algorithm was used to evaluate the efficiency of immunotherapy (Jiang et al. 2018). The higher score was observed in C2 subtype, suggesting that the C2 subtype had a greater possibility of immune escape and the possibility of benefiting from immunotherapy.

Difference analysis comparing C2 subtypes with C1 subtypes filtered a total of 786 shared differential genes in the TCGA and GSE cohorts. Further univariate Cox and LASSO regression analysis identified seven hypoxia-related signature genes, including UQCRFS1, KRAS, KLF4, HOXA5, GMPR, ISG20, and SNRPD1. Next, HYRS model was built to predict the prognosis of OC. The UQCRFS1 protein belongs to complex III of the mitochondrial respiratory chain, and was reported to participate in regulating OC development (Kaneko et al. 2003). Ha et al. showed that the expression level of UQCRFS1 was significantly increased in advanced OC, indicating that high levels of UQCRFS1 predict poorer prognosis (Ha et al. 2021). In contrast, KLF4 is down-regulated in OC (Ma et al. 2019). Up-regulation of KLF4 can enhance the therapeutic effect of chemotherapeutic drugs on OC by affecting cancer cell proliferation (Ma et al. 2019 ). KRAS transmits signals from the extracellular to the nucleus. KRAS is a member of the RAS/MAPK signaling pathway, and its main role is to participate in the regulation of cell proliferation and differentiation (McCormick 2015). In OC, KRAS served as a biomarker and potential therapeutic targets have been reported (Ratner et al. 2010; Ratner et al. 2010). Notably, KAS is a proto-oncogene (Siddiqui-Jain et al. 2002). KLF4 was a tumor suppressor in ovarian cancer cells by inhibiting the epithelial to mesenchymal transition (Wang et al. 2017b). Yu et al. have constructed a five glycolysis‐related genes signature, including ISG20, for patients with OC (Yu et al. 2021). Lower SNRPD1 expression indicated poorer outcome of OV (Bao et al. 2020). Above findings suggested the efficiency of our HYRS model. Pathway analysis showed the hypoxia pathway was activated in the high HYRS group both in TCGA and GSE cohorts. Hypoxia is closely associated with tumor progression, which appears to be one of the reasons for the lower survival in the high HYRS group (Jing et al. 2019). This study performed molecular subtyping of OC according to the hypoxia gene expression signature and developed a HYRS model with excellent predictive efficiency, but the pathogenesis of OC still requires further validation due to the complexity of the actual situation.

Moreover, we compared the HYRS model with the TIDE algorithm to evaluate the effectiveness of the HYRS model in predicting prognosis. The results showed that HYRS had a high predictive efficiency.

There were some limitations in this study. Firstly, it was necessary to verify the significance of hub genes in cancer tissues through experiments, such as RT-qPCR, IHC, and Western blot. Secondly, although our results showed a strong predictive potential and clinical value of the prognostic signature, prospective studies were needed to demonstrate the clinical application and prognostic value of the model in patients.

## Conclusion

In short, OC was classified into two molecular subtypes based on hypoxia-related gene expression signatures. Further, seven hypoxia-related OC prognosis-related signature genes, including UQCRFS1, KRAS, KLF4, HOXA5, GMPR, ISG20, and SNRPD1, were obtained as a whole new combination. Significantly, the HYRS model could predict OC prognosis effectively.

## Supplementary Information

Below is the link to the electronic supplementary material.

Supplementary file1 (DOCX 17 KB)

## Data Availability

The datasets generated and/or analyzed during the current study are available in the [GSE26712] repository [https://www.ncbi.nlm.nih.gov/geo/query/acc.cgi?acc=GSE26712]; in [GSE91061] repository [https://www.ncbi.nlm.nih.gov/geo/query/acc.cgi?acc=GSE91061].

## Abbreviations

TIME: Tumor immune microenvironment
OC: Ovarian cancer
HYRS: Hypoxia-related risk score model
TCGA: The Cancer Genome Atlas
LASSO: Least absolute shrinkage and selection operator
ROC: Receiver operating characteristic
DCA: Decision curve analysis
GSEA: Gene set enrichment analysis
OS: Overall survival
ROS: Reactive oxygen species
CDF: Cumulative distribution function
TIDE: Tumor immune dysfunction and exclusion
ESTIMATE: Estimation of STromal and Immune cells in MAlignant Tumors using Expression data
ICI: Immune checkpoint inhibitors
KM: Kaplan–Meier
MCP-Counter: Microenvironment cell populations-counter
DEGs: Differentially expressed genes
