# Supplementary material for: Identification of seven hypoxia-related genes signature and risk score models for predicting prognosis for ovarian cancer
Source: Funct Integr Genomics. 2023 Jan 16;23(1):39. doi: 10.1007/s10142-022-00956-3 (PMC9841006; doi:10.1007/s10142-022-00956-3)
Supplement: Supplementary file 1 — Supplementary file1 (DOCX 17 KB) [file 10142_2022_956_MOESM1_ESM.docx]

Table S1. prognostic genes in univariate regression Cox analysis

| genes | p-value | HR | Low 95%CI | High 95%CI |
| --- | --- | --- | --- | --- |
| AKAP12 | 0.02 | 1.12 | 1.01 | 1.25 |
| ANGPTL4 | 0.02 | 1.12 | 1.01 | 1.24 |
| ANXA2 | 0.06 | 1.20 | 0.98 | 1.46 |
| CDKN1B | 0.02 | 1.23 | 1.02 | 1.48 |
| CITED2 | 0.005 | 1.21 | 1.05 | 1.40 |
| CXCR4 | 0.01 | 0.85 | 0.75 | 0.97 |
| DDIT4 | 0.01 | 1.13 | 1.02 | 1.26 |
| DUSP1 | 0.03 | 1.10 | 1.00 | 1.20 |
| PPP1R15A | 0.02 | 1.20 | 1.02 | 1.41 |
| PPP1R3C | 0.02 | 1.17 | 1.02 | 1.34 |
| PRDX5 | 0.01 | 0.73 | 0.57 | 0.95 |
| SLC2A3 | 0.03 | 1.13 | 1.01 | 1.26 |
| ZFP36 | 0.04 | 1.10 | 1.00 | 1.21 |
| ISG20 | 0.01 | 0.84 | 0.72 | 0.97 |
